# ‘Leaving no stones unturned’: up to 10 years results on the effectiveness, tolerability and metabolic safety of dolutegravir+lamivudine (DTG+3TC) as a switch regimen in the ODOACRE cohort

**DOI:** 10.1093/jac/dkag172

**Published:** 2026-05-15

**Authors:** Arturo Ciccullo, Gianmaria Baldin, Adriana Cervo, Letizia Oreni, Maria Mazzitelli, Giuseppe Gasparro, Marianna Menozzi, Mario Cesaretti, Francesco Bassani, Filippo Lagi, Francesca Lombardi, Andrea Giacomelli, Alberto Borghetti, Massimiliano Fabbiani, Stefano Rusconi, Annamaria Cattelan, Spinello Antinori, Cristina Mussini, Simona Di Giambenedetto

**Affiliations:** Infectious Diseases Unit, Ospedale San Salvatore, L’Aquila, Italy; Fondazione Policlinico Universitario A. Gemelli IRCCS, UOC Malattie Infettive, Roma, Italia; Department of Infectious Diseases, Azienda Ospedaliero-Universitaria of Modena, Modena, Italy; Department of Infectious Diseases, ASST Fatebenefratelli Sacco, Luigi Sacco Hospital, Milan, Italy; Fondazione Policlinico Universitario A. Gemelli IRCCS, UOC Malattie Infettive, Roma, Italia; Infectious and Tropical Diseases Unit, Padua University Hospital, Padua, Italy; Dipartimento di Sicurezza e Bioetica, Università Cattolica del Sacro Cuore, Roma, Italia; Department of Experimental and Clinical Medicine, University of Florence, Florence, Italy; Department of Infectious Diseases, Azienda Ospedaliero-Universitaria of Modena, Modena, Italy; Infectious Diseases Unit, Department of Clinical and Experimental Medicine, Azienda Ospedaliera Universitaria Pisana, Pisa, Italy; Infectious Diseases Unit, Ospedale Civile di Legnano ASST Ovest Milanese, Legnano, Italy; Department of Experimental and Clinical Medicine, University of Florence, Florence, Italy; Fondazione Policlinico Universitario A. Gemelli IRCCS, UOC Malattie Infettive, Roma, Italia; Dipartimento di Sicurezza e Bioetica, Università Cattolica del Sacro Cuore, Roma, Italia; Department of Infectious Diseases, ASST Fatebenefratelli Sacco, Luigi Sacco Hospital, Milan, Italy; Department of Biomedical and Clinical Sciences, Università Degli Studi di Milano, Milan, Italy; Infectious Diseases Unit, Department of Clinical and Experimental Medicine, Azienda Ospedaliera Universitaria Pisana, Pisa, Italy; Department of Medical Sciences, Infectious and Tropical Diseases Unit, University Hospital of Siena, Siena, Italy; Infectious Diseases Unit, Ospedale Civile di Legnano ASST Ovest Milanese, Legnano, Italy; Department of Biomedical and Clinical Sciences, Università Degli Studi di Milano, Milan, Italy; Infectious and Tropical Diseases Unit, Padua University Hospital, Padua, Italy; Department of Biomedical and Clinical Sciences, Università Degli Studi di Milano, Milan, Italy; III Infectious Diseases Unit, ASST Fatebenefratelli Sacco, Luigi Sacco Hospital, Milan, Italy; Department of Infectious Diseases, Azienda Ospedaliero-Universitaria of Modena, Modena, Italy; Fondazione Policlinico Universitario A. Gemelli IRCCS, UOC Malattie Infettive, Roma, Italia; Dipartimento di Sicurezza e Bioetica, Università Cattolica del Sacro Cuore, Roma, Italia

## Abstract

**Background:**

Dolutegravir plus lamivudine (DTG+3TC) is widely used as a two-drug switch regimen for virologically suppressed PWH. We report long-term real-world data on effectiveness, tolerability, immunological recovery and metabolic/cardiovascular outcomes in a large, multicentre Italian cohort.

**Methods:**

We performed a retrospective observational analysis of participants in the ODOACRE cohort who switched to DTG+3TC between January 2015 and January 2025 across eight Italian centres. Inclusion criteria were age ≥18, HIV-RNA <50 copies/mL for ≥6 months at switch, HBsAg negative. Primary outcomes were time to virological failure (VF) and time to treatment discontinuation (TD) for any cause. Kaplan–Meier survival analysis and Cox regression models were used to evaluate predictors. Changes in metabolic and immunological markers were assessed using linear mixed models and linear regression.

**Results:**

A total of 2535 participants were included. Estimated probabilities of maintaining virological suppression were 99.5% at 48 weeks, 96.1% at 240 weeks and 90.4% at 480 weeks. In multivariate analysis, longer time since HIV diagnosis (per year aHR 1.038) and zenith HIV-RNA >500 000 copies/mL (aHR 2.205) predicted VF, whereas longer prior virological suppression was protective (per year aHR 0.869). During 9721.6 PYFU we observed 362 TDs (3.72 per 100 PYFU), with probabilities of regimen maintenance of 94.4%, 83.5% and 78.7% at weeks 48, 240 and 480, respectively.

**Conclusions:**

In our real-world cohort with extended follow-up, DTG+3TC as a switch regimen was associated with durable virological suppression and favourable tolerability. Metabolic findings should be considered descriptive and exploratory, within the limits of an observational, uncontrolled study.

## Background

The introduction of integrase strand-transfer inhibitor (INSTI) transformed HIV treatment, providing People Living with HIV (PWH) an antiretroviral class with high efficacy, high genetic barrier and improved tolerability.^1^ Among them, dolutegravir, the first second-generation INSTI, combines a higher genetic barrier than first-generation INSTIs with once-daily dosing and no pharmacological boosters, making it ideal for 3-drug and 2-drug regimens (2DR), without compromising potency. Its combination with lamivudine has become widely prescribed in switch regimens, demonstrating efficacy, safety and minimal metabolic impact.^2–5^ Recently, this combination, now available as a single tablet regimen (STR) has also been studied as a first-line regimen in people starting antiretroviral therapy (ART), confirming its non-inferiority compared to other INI-based 3DR.^6,7^

Since 2015, we have analysed data on PWH switching to dolutegravir+lamivudine in routine care, with a follow-up duration often exceeding those reported in clinical trials. Building on more than a decade of clinical experience, we present an updated analysis with extended follow-up. Given growing interest in cardiovascular disease (CVD) in PWH, and since emerging reports suggest that INIs may increase CVD risk,^8,9^ we also evaluated CVD risk and included markers of insulin resistance as proxies for metabolic syndrome, to provide a comprehensive view of sustained clinical outcomes in virologically suppressed, treatment-experienced people receiving dolutegravir+lamivudine.

## Materials and methods

### Study design

This retrospective observational study enrolled virologically-suppressed PWH from eight Italian centres participating in the ODOACRE cohort^10^ who switched to dolutegravir+lamivudine between January 2015 and January 2025. Follow-up was calculated from the switch date (baseline) until treatment discontinuation, death or last available visit, whichever occurred first, without formal collection of loss to follow-up. Both 2-pill regimen and STR (available in Italy since 2019) were included. Inclusion criteria were: informed consent to data collection and study participation, age ≥ 18 years, stable viral suppression at baseline (HIV-RNA < 50 copies/mL for ≥6 months) and HBsAg negative.

Clinical data collected included history, Body Mass Index (BMI), serum lipid and glycaemic markers (blood glucose, total/HDL cholesterol ratio, triglycerides), immunological (absolute and percentage CD4+ T-cell counts, CD4/CD8 ratio), and virological markers; we also calculated SCORE-2, estimating 10-years cardiovascular risk, and METS-IR, estimating glucose intolerance. Missing data were handled using an available-case approach, and analyses were restricted to participants with available measurements for the specific parameter at each time point: analyses of metabolic parameters were performed in the subset of individuals with available data (BMI *n* = 777, SCORE-2 *n* = 307, METS-IR *n* = 543). Major cardiovascular events, pregnancies, and malignancies during follow-up were also recorded.

Primary study objectives were to evaluate time to virological failure (VF, defined as a single HIV-1 RNA ≥1000 copies/mL or two consecutive HIV-1 RNA ≥50 copies/mL) and time to treatment discontinuation (TD, defined as interruption of either lamivudine or dolutegravir for any reason). Secondary objectives included the assessment of factors associated with TD, immunological changes, longitudinal changes in metabolic parameters (BMI, lipid profile, SCORE2, METS-IR) and the incidence of major clinical events, including cardiovascular events (such as myocardial infarction or stroke) and malignancies. Survival analyses were performed to evaluate time to VF and time to TD, using Kaplan–Meier estimates and Cox regression models to identify potential predictors. Variables were selected for multivariable models based on clinical relevance and results from univariate analyses. To reduce the risk of overfitting, only a limited number of covariates were included in the final models, taking into account the number of observed events. Longitudinal changes in metabolic and cardiovascular parameters were assessed using linear mixed-effects models for repeated measures; linear regression analyses explored associated variables. Two-sided *P* < 0.05 was considered significant. Analyses were performed using SPSS 23.0 (IBM Corp.). The study adhered to the Declaration of Helsinki and was approved by institutional ethics committees.

## Results

### Study population

Overall, 2535 people were included: 1651 (65.1%) were males, with a median age of 55 years (Interquartile Range [IQR] 46–62). Median time from HIV diagnosis was 15.2 years (IQR 8.9–23.7), while median time on ART before switching to dolutegravir+lamivudine was 11.5 years (IQR 6.0–18.3). Median follow-up duration after switching was 3.54 years (IQR 1.98–5.50), accounting for a total of 9721.6 person-years of follow-up. Five hundred and seventeen (among 2386 who were tested, 21.7%) had detectable HBc antibodies and 383 (15.1%) anti-HCV antibodies. Out of 2052 people who had a previous genotypic resistance test available, 103 (5.0%) had the M184 V resistance mutation. Full population characteristics are shown in Table 1.

**Table 1.** Population characteristics at baseline

Variables	*n* = 2535
Age (years), Median (IQR)	55 (46–62)
Male sex at birth, *n* (%)	1651 (65.1)
Risk factor for HIV infection, *n* (%):	
Heterosexual	970 (38.3)
MSM	1043 (41.1)
IDU	271 (10.7)
Others	251 (9.9)
Anti-HBc antibodies positive, *n* (%)	517/2386 (21.7)
Anti-HCV antibodies positive, *n* (%)	383 (15.1)
Time from HIV diagnosis (years), Median (IQR)	15.2 (8.9–23.7)
CDC stage C, *n* (%)	484 (19.1)
Time on antiretroviral therapy (years), Median (IQR)	11.5 (6.0–18.3)
M184V at a previous genotypic exam, *n* (%)	103/2052 (5.0)
Nadir of CD4+ (cell/µL), Median (IQR)	250 (118–374)
Zenith HIV-RNA (log copies/mL), Median (IQR)	4.95 (4.42–5.46)
Zenith HIV-RNA > 500.000 copies/mL, *n* (%)	313 (12.3)
Previous virological failure, *n* (%)	488 (19.2)
CD4+ count (cell/µL) at baseline, Median (IQR)	701 (537–915)
CD4/CD8 ratio at baseline, Median (IQR)	0.88 (0.61–1.24)
Time of virological suppression (years), Median (IQR)	8.8 (4.3–14.0)
Previous ART regimen, *n* (%):	
2NRTI+INI	1027 (40.5)
2NRTI+NNRTI	554 (21.9)
2NRTI+PI	219 (8.6)
3TC-based 2DR	325 (12.8)
Other/Unknown	410 (16.2)
DTG in previous regimen, *n* (%)	599 (23.6)
Reasons for switch, *n* (%):	
Simplification	1764 (69.6)
Dyslipidaemia/Cardiovascular risk	127 (5.0)
Gastrointestinal or liver toxicity	55 (2.2)
Renal toxicity	31 (1.2)
Osteoporosis	24 (0.9)
Neurological toxicity	7 (0.3)
Other toxicities	49 (1.9)
Drug-drug interactions	54 (2.1)
Hypersensitivity reaction	3 (0.1)
Other/Unknown reasons	421 (16.6)
Total cholesterol at baseline, median (IQR)	193 (168–221)
LDL cholesterol at baseline, median (IQR)	117 (96–142)
HDL cholesterol at baseline, median (IQR)	48 (40–59)
Triglycerides at baseline, median (IQR)	111 (80–162)
SCORE2 at baseline, median (IQR)	4.9 (2.8–7.3)
METS-IR at baseline, median (IQR)	37.5 (32.2–42.8)
Body Mass Index at baseline, median (IQR)	24.9 (22.5–27.5)

### Virological efficacy and safety

During 9627.1 PYFU, we observed 89 VF (0.92 per 100 PYFU), with 49 (55.0%) occurring with a HIV-RNA >1000 copies/mL. All individuals who experienced VF regained virological suppression in the following 12 months, with 21 (23.6%) maintaining dolutegravir+lamivudine.

**Figure 1.**
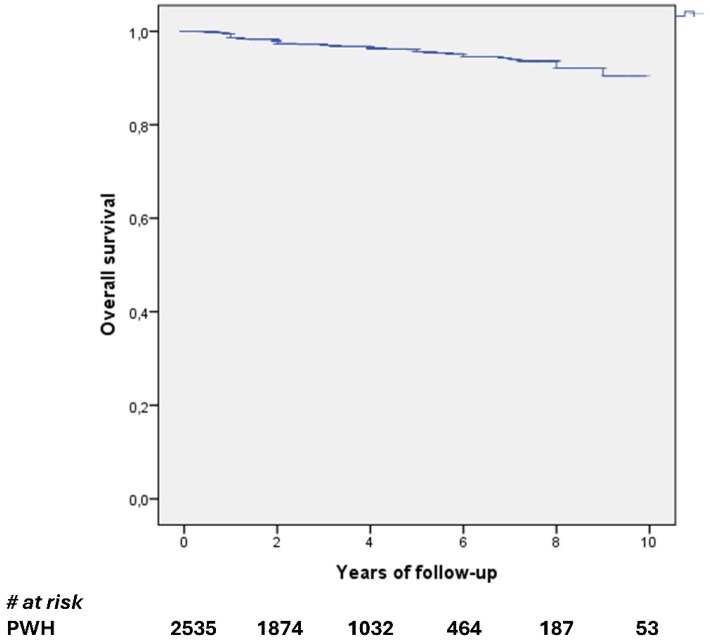
Survival analysis for virological failure.

Estimated probabilities of maintaining virological suppression at 48, 240 and 480 weeks were 99.5% (SD ± 0.1), 96.1% (SD ± 0.5), and 90.4% (SD ± 2.1), respectively (Figure 1).

At multivariate analysis, a longer time since HIV diagnosis (per 1 year more, aHR 1.038, 95%CI 1.006 to 1.072, *P* = 0.019) and a zenith HIV-RNA >500 000 cps/mL (aHR 2.205, 95%CI 1.250 to 3.887, *P* = 0.006) were significantly associated with a higher risk of VF. By contrast, a longer time of virological suppression (per 1 year longer, aHR 0.869, 95%CI 0.821 to 0.919, *P* < 0.001) resulted inversely correlated with VF risk, after adjusting for the presence of M184 V mutation, past HBV and HCV infection, and CDC stage [Table 2].

**Table 2.** Multivariate Cox regression analysis for predictors of virological failure

Variable	aHR	95% CI	*P*
Positive anti-HBc antibodies	1.340	0.705–2.548	0.372
Positive anti-HCV antibodies	1.161	0.560–2.405	0.688
CDC stage C	0.959	0.521–1.767	0.894
History of M184V	1.566	0.646–3.798	0.321
HIV-RNA Zenith over 500’000 cps/mL	2.205	1.250–3.887	0.006
Years of virological suppression	0.869	0.821–0.919	<0.001
Years of HIV infection	1.038	1.006–1.072	0.019

Since time spent on virological suppression was a significant predictor of VF, we further expanded our analysis and found that people with a virological suppression lasting 8 years or longer had a significant higher probability of maintaining virological suppression during follow-up (96.6% at week 240% and 91.5% at week 480), compared who those with a shorter duration (94.1% at week 240% and 88.3% at week 480, log-rank *P* = 0.004). Similarly, people with a zenith HIV-RNA lower than 500 000 cps/mL had a higher probability of maintaining virological suppression (94.9% at week 240% and 91.8% at week 480) compared with those with a higher than 500 000 cps/mL zenith HIV-RNA (92.5% at week 240% and 62.2% at week 480, log-rank *P* = 0.023).

At time of failure, only 12/89 (13.5%) people had a genotypic resistance testing done after VF, with no evidence of emerging resistance mutations either to lamivudine or dolutegravir.

### Treatment tolerability

In our cohort, during 9721.6 PYFU, we observed 362 TD (3.72 per 100 PYFU). Reasons for discontinuing were: toxicity (82 cases, 22.6% of total TD), switch to a long-acting injectable regimen (67, 18.5%), treatment intensification (38, 10.5%), switch to a STR (30, 8.3%), death (8, 2.2%), weight gain (6, 1.7%), pregnancy (6, 1.7%), drug-drug interaction (4, 1.1%), other/unknown (121, 33.4%). Among individuals with HBcAb positivity at baseline, no cases of HBV reactivation, HBsAg seroconversion, or clinically significant hepatic flares were observed during follow-up, and no treatment discontinuations due to HBV-related hepatic toxicity occurred.

**Figure 2.**
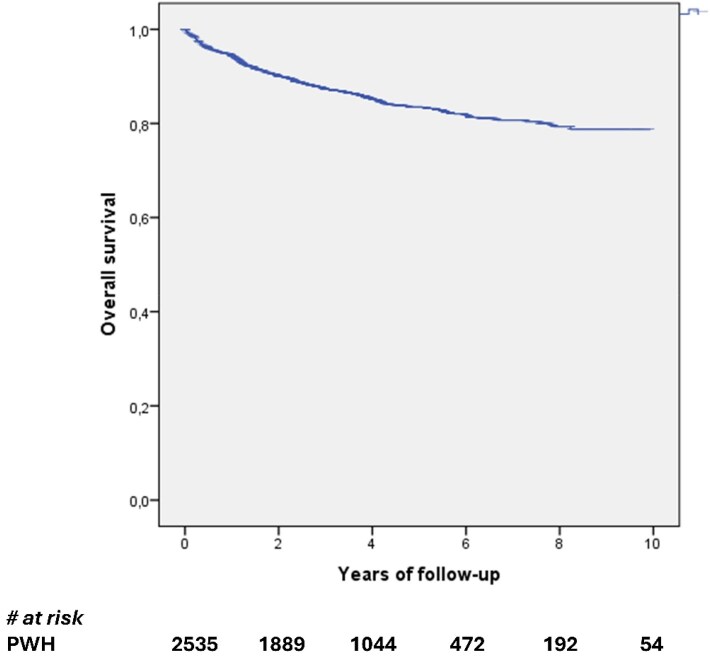
Survival analysis for treatment discontinuation.

Estimated probabilities of maintaining study regimen at 48, 240 and 480 weeks were 94.4% (SD ± 0.5), 83.5% (SD ± 0.9) and 78.7% (SD ± 1.4), respectively (Figure 2).

At the multivariate analysis, switching from a dolutegravir-containing regimen (aHR 0.474, 95%CI 0.317 to 0.707, *P* < 0.001) was associated with a lower risk of TD, while a zenith HIV-RNA over 500 000 cps/mL (aHR 1.44, 95%CI 1.068 to 1.955, *P* = 0.017) was associated with a higher risk of TD after adjusting for HCV coinfection and years since HIV diagnosis [Table 3].

**Table 3.** Multivariate Cox regression analysis for predictors of treatment discontinuation

Variable	aHR	95% CI	*P*
Dolutegravir in previous ART regimen	0.474	0.317–0.707	<0.001
HIV-RNA Zenith over 500’000 cps/mL	1.445	1.068–1.955	0.017
Positive anti-HCV antibodies	1.265	0.908–1.762	0.164
Years of ART exposure	0.984	0.966–1.002	0.074

In a dedicated subanalysis, we found that people coming from a dolutegravir-containing regimen had a significantly higher probability of maintaining dolutegravir+lamivudine (87.9% at week 240 and 85.0 at week 480) compared to those not exposed to dolutegravir in the previous ART regimens (82.2% at week 240% and 77.4% at week 480, log-rank *P* < 0.001).

Regarding discontinuations due to toxicity: 27 people discontinued due to CNS toxicity (1.1% of total population), 11 (0.4%) due to gastrointestinal toxicity, 7 (0.3%) due to renal toxicity and 37 (1.5%) due to other/unspecified toxicity. In our cohort, the vast majority of discontinuations (54/82 65.6%) occurred in the first year of follow-up.

Probability of discontinuation following neuropsychiatric events was 6.4% at week 48% and 13.8% at weeks 240 and 480. Female sex at birth (versus male sex, aHR 3.57, *P* = 0.006) and HCV-coinfection (aHR 2.99, *P* = 0.024) were significantly associated with discontinuations due to CNS toxicity, after adjusting for age and zenith HIV-RNA.

### Immunological assessment

CD4+ T-cell count continued to significantly increase during the study follow-up (median change +31 cell/mm^3^ at 48 weeks, *P* < 0.001; +46 cell/mm^3^at week 144, *P* < 0.001; +53 cell/mm^3^ at week 240, *P* < 0.001; and +135 cell/mm^3^ at week 480, *P* < 0.001).

At the multivariate analysis both age (per 10 years more, B −24.5, 95%CI −41.4 to −7.6, *P* = 0.005) and baseline CD4+ T-cell count (per 10 cell/mm^3^ more, B −2.4, 95%CI −3.0 to −1.7, *P* < 0.001) negatively predicted an improvement in CD4+ T-cell count at 240 weeks; meanwhile CD4+ T-cell count nadir (per 10 cell/mm^3^ more, B 3.2, 95%CI 2.0 to 4.5, *P* < 0.001) was associated with a more pronounced improvement after adjusting for peak CDC stage, M184 V status and the presence of HBcAb.

We also observed a significant increase in CD4/CD8 ratio after 144 (+0.04, *P* = 0.007) and 240 weeks of follow-up (+0.1, *P* < 0.001). The trend was confirmed in people with data at 480 weeks of follow-up, with a median change in CD4/CD8 ratio of +0.22, although not statistically significant (*P* = 0.327). No predictors of change were found in our analysis.

Evaluating the proportion of people achieving a CD4/CD8 ratio ≥1, this was significantly higher both at 144 and 240 weeks of follow-up compared with baseline values; in particular, CD4/CD8 ratio was ≥1 in 40.4% of people at baseline, in 53.4% at 144 weeks (*P* < 0.001) and in 57.3% at 240 weeks (*P* < 0.001).

### Metabolic profile and cardiovascular risk

Analysing changes in CVD-risk, we performed SCORE-2 and METS-IR at different follow-up time points. We observed an increase in SCORE-2 at 48 and 144 weeks, with median increases of +0.1 (*P* = 0.002) and +0.5 (*P* < 0.001), respectively. SCORE-2 increase at 48 weeks was positively predicted by older age (per 10 years more, B 0.47, 95%CI 0.05 to 0.90, *P* = 0.029) after adjusting for baseline SCORE-2, sex, baseline BMI, years of HIV infection, years of ART exposure and previous ART regimen. SCORE-2 changes at week 144 were similarly positively predicted by older age (per 10 years more, B 1.52, 95%CI 0.84 to 2.20, *P* < 0.001) and inversely correlated with baseline SCORE-2 (per each unit higher, B −0.08, 95%CI −0.16 to 0.01, *P* = 0.002) using the same regression model.

Regarding METS-IR, we observed a significant decrease at 48 weeks (−0.3, *P* = 0.05) and 144 weeks (−1.0, *P* = 0.023). Decrease in METS-IR after 144 weeks was more pronounced in people with a higher METS-IR score at baseline (per each unit higher, B −0.23, 95%CI −0.30 to −0.17, *P* < 0.001) and in younger persons (per 10 years older, B 0.64, 95%CI 0.13 to 1.16, *P* = 0.015) after adjusting for sex and previous ART regimen.

As to serum lipid markers, after 240 weeks we registered a significant decrease in total cholesterol (*P* < 0.001), triglycerides (*P* < 0.001), LDL cholesterol (*P* < 0.001) as well as an improvement in HDL cholesterol (*P* = 0.027). Similarly, after 480 weeks, we found significant reduction in total cholesterol (*P* < 0.001) and triglycerides (*P* < 0.001) and increase in HDL cholesterol (*P* < 0.001). Median changes are shown in Table 4.

**Table 4.** Changes in blood metabolic parameters during follow-up

Variable	Median at BL (IQR)	Median at week 240	*P* at week 240	Median at week 480	*P* at week 480
Total cholesterol (mg/dL)	193 (168–221)	187 (158–211)	<0.001	182 (156–213)	<0.001
LDL cholesterol (mg/dL)	117 (96–142)	108 (88–133)	<0.001	105 (81–145)	0.250
HDL cholesterol (mg/dL)	48 (40–59)	48 (40–58)	0.012	50 (42–62)	0.001
Triglycerides (mg/dL)	111 (80–161)	104 (76–148)	<0.001	98 (76–138)	<0.001

In linear regression analysis, higher baseline cholesterol levels were significantly associated with a greater reduction in cholesterol after 240 weeks (t −3.4, *P* = 0.001); similarly higher baseline triglycerides were associated with a more pronounced decrease in triglycerides after 240 weeks (t −9.4, *P* < 0.001) and higher LDL levels at baseline were associated with a more pronounced decrease in LDL levels after 240 weeks (t −4.6, *P* < 0.001). No predictors of change were found for HDL.

Finally, in our cohort, we did not observe significant changes in BMI during follow-up. In particular, with median baseline BMI being 24.9, after 144 and 240 weeks of follow-up, median BMI was 24.9 (*P* = 0.942) and 25.6 (*P* = 0.504), respectively.

### Major clinical events

During follow-up, 33 (1.3%) people experienced a major cardiovascular event, with 25 of them diagnosed with myocardial infarction and 5 with ictus cerebri. Moreover, 54 (2.1%) were diagnosed with one malignancy (Table S1, available as Supplementary data at *JAC* Online). Considering both cardiovascular events and malignancies as major clinical events, we observed that the probability of remaining free from major clinical events in our cohort was 95.8% (SD ± 0.5) and 91.1% (SD ± 1.4) at weeks 240 and 480, respectively. At a multivariate analysis, age (aHR 1.05, *P* < 0.001), and years living with HIV (aHR 1.06, *P* < 0.001) were associated with a higher risk of major clinical event after adjusting for sex, years of ART exposure, zenith HIV-RNA and CD4+ T-cell count at nadir.

Considering only myocardial infarction [MI], we observed in our cohort a rate of 2.59 (±1.02) events per 1000 PYFU. In a multivariate analysis, longer time from HIV diagnosis (aHR 1.086, 95%CI 1.025 to 1.150, *P* = 0.005) and a zenith HIV-RNA over 500 000 cps/mL (aHR 3.279, 95%CI 1.261 to 8.527, *P* = 0.015) predicted MI after adjusting for age, sex at birth, CD4+ cell nadir and total cholesterol levels at baseline.

Finally, two women had a pregnancy during follow-up. Both continued the study regimen with a favourable pregnancy outcome and no complications.

## Discussion

To date, our study provides one of the longest real-world follow-ups describing the effectiveness, safety, tolerability and metabolic impact of dolutegravir+lamivudine as a switch strategy. While randomized trials have established short- and medium-term efficacy of this regimen, long-term observational cohorts allow exploration of scenarios rarely captured in trials, including ageing with HIV, treatment adherence, comorbidities and regimen modifications. Compared with previous reports from the same cohort, the present analysis provides substantially longer follow-up, allowing the assessment of long-term durability of virological suppression up to 10 years, as well as the evaluation of late outcomes that are typically not captured in clinical trials or shorter observational studies, including long-term treatment persistence, cardiometabolic trajectories and major clinical events, extending previous cohort observations substantially.

In our cohort, individuals switching to dolutegravir+lamivudine showed a 90.4% probability of maintaining virological suppression after 480 weeks, among the longest durability assessments reported in clinical practice.^2–4,11^

Higher zenith HIV-RNA and longer time living with HIV were associated with increased VF risk, consistent with previous reports.^12,13^ These factors may reflect a higher viral burden in long-lived reservoirs, potentially contributing to incomplete virologic control. Nevertheless, the VF rate remained low (0.92/100 PYFU). Among the subset of individuals who underwent genotypic resistance testing after confirmed VF, no emergent resistance mutations to either dolutegravir or lamivudine were detected. However, the limited number of tests substantially restricts any conclusions on resistance emergence, and findings should be interpreted with caution despite consistency with previous reports.

Consistent with previous findings,^4^ the presence of M184 V was not associated with an increased risk of VF, although the small number of individuals with this mutation should be carefully considered when interpreting this finding. Earlier studies suggested increased VF risk in individuals with M184 V and shorter durations of prior virological suppression.^12,14^ In contrast, in our analysis, where duration of virological suppression was included in the model, no association emerged. These results align with other real-world studies reporting effectiveness of dolutegravir+lamivudine even in the presence of historical mutations affecting 3TC activity,^15^ although the small number of individuals with M184 V in our cohort and the observational design limit the strength of this finding. Similarly, although concerns have been raised regarding the use of two-drug regimens in individuals with previous hepatitis B virus infection,^16^ we did not observe reduced virological effectiveness in PWH with isolated anti-HBc positivity. No cases of HBV reactivation or clinically significant hepatic flares were observed during follow-up, and no discontinuations were attributed to HBV-related toxicity. However, HBV-DNA monitoring was not standardized across centres.

TD occurred in 362 individuals (14.3%), corresponding to a 78.7% probability of continuation at 480 weeks. Simplification accounted for 26.8% of discontinuations, often towards STR or long-acting strategies. Since dolutegravir/lamivudine STR became widely available in Italy only in 2020, switches towards STR before that time required alternative combinations. Prior dolutegravir exposure reduced discontinuation risk, possibly reflecting early onset of dolutegravir-related adverse events.^17,18^ This finding should be interpreted in light of potential survivor bias, as individuals remaining on treatment longer are those who tolerated therapy.

Toxicity-related discontinuation occurred in 82 individuals, with 27 (1.1%) stopping due to CNS events. Following early reports describing neuropsychiatric toxicity associated with dolutegravir,^19^ clinicians often adopted a cautious approach and sometimes discontinued therapy at the onset of mild symptoms. Nevertheless, our 1.1% rate is lower than the 5%–6% reported elsewhere.^19–21^ Women and individuals with past HCV infection were at higher risk of CNS-related discontinuation, confirming previous observations.^4,20,22^

CD4+ cell count and CD4/CD8 ratio improved during follow-up, supporting sustained immunological stability with prolonged use of dolutegravir+lamivudine.

Regarding metabolic outcomes, switching to dolutegravir+lamivudine showed reductions in total cholesterol, triglycerides and LDL cholesterol, while HDL cholesterol increased during follow-up, although these findings should be considered exploratory. These results are consistent with previous studies reporting a favourable lipid profile,^2,4^ although causal interpretation cannot be inferred. Greater lipid reductions among PWH with higher baseline levels should be interpreted cautiously, as they may reflect initiation of lipid-lowering therapy. The REPRIEVE Trial^23^ has increased attention to cardiovascular prevention in PWH, potentially promoting broader statin use. Because information on lipid-lowering therapy and lifestyle factors was not systematically captured, residual confounding cannot be excluded.

SCORE-2 increased during follow-up, particularly among older individuals. This finding is not unexpected, as chronological age is one of the main contributors to estimated 10-year cardiovascular risk, and the observed increase likely reflects the progressive ageing of the cohort rather than a direct treatment effect.^24^

In contrast, METS-IR decreased at weeks 48 and 144, particularly among individuals with higher baseline values. These findings should be interpreted with caution and considered exploratory, potentially influenced by unmeasured confounders; moreover, METS-IR has not been specifically validated in PWH and may be influenced by unmeasured factors, as lifestyle and concomitant therapy data were unavailable. Nonetheless, this signal may be of interest and warrants further investigation in dedicated studies. Insulin sensitivity is a key determinant of metabolic homeostasis and a major driver of metabolic syndrome in the general population and in PWH^25^ and METS-IR has been associated in large population cohorts with cardiovascular and all-cause mortality,^26^ underscoring its relevance as a complementary cardiometabolic marker in ART switch assessments.

BMI remained largely stable throughout follow-up, suggesting that the reduction in METS-IR was unlikely to be primarily driven by major changes in body weight. Instead, these improvements may partly reflect the metabolic impact of discontinuing previous antiretroviral regimens with less favourable metabolic profiles. Concerns regarding INI-associated weight gain have grown,^27^ given links between weight gain, metabolic syndrome, and mortality.^28^ However, in line with findings from the PASO-DOBLE trial,^29^ our data did not show significant weight increases after switching to dolutegravir+lamivudine.

MI incidence was 2.59 events per 1000 PYFU, consistent with rates reported in European cohorts of PWH, which typically range between 2–5 events per 1000 PYFU, reflecting the well-established elevated cardiovascular risk in this population.^30^ In multivariate analysis, a longer duration since HIV diagnosis and zenith HIV-RNA >500 000 copies/mL were independently associated with MI. These factors may reflect the cumulative impact of long-standing HIV infection, immune activation and chronic inflammation on vascular health.^30^ However, our study was not specifically designed to evaluate cardiovascular outcomes. The number of events was modest, residual confounding cannot be excluded, and detailed longitudinal data on traditional cardiovascular risk factors were not available.

This study has several limitations. First, its retrospective observational design, heterogeneous follow-up and the absence of a comparator group limit causal inference; findings should be interpreted as descriptive, within-cohort observations. Second, we were not able to systematically capture the initiation of lipid-lowering, antidiabetic, or weight-management medications, nor lifestyle factors during follow-up, which may have influenced the metabolic parameters observed over time. Third, several analyses, including SCORE-2, METS-IR and BMI changes, were performed in subsets of participants with available data, which may limit the generalizability of these findings and introduce residual confounding. Also, changes in METS-IR should be interpreted cautiously because this index has not been specifically validated in PWH and may be influenced by unmeasured confounders. Fourth, the number of cardiovascular events was relatively modest, and multivariable analyses should therefore be considered exploratory and hypothesis-generating, particularly at longer follow-up timepoints. Fifth, given the long follow-up, attrition over time may have introduced survivor bias, and detailed follow-up distribution was not available. Finally, genotypic resistance testing was available only in a limited proportion of individuals experiencing VF, and the small number of participants with M184 V may have reduced our ability to fully characterize resistance emergence. Nevertheless, the large multicentre cohort and the extended duration of follow-up provide valuable real-world evidence on the long-term durability, safety and clinical outcomes associated with dolutegravir+lamivudine in routine clinical care.

### Conclusions

In this large, multicentre real-world cohort, switching to dolutegravir+lamivudine was associated with sustained virological suppression, good tolerability and no clear detrimental metabolic trends over prolonged follow-up. The regimen showed a low rate of discontinuation and no evidence of emergent resistance in the limited number of tested cases. No clear detrimental effects were observed in lipid profile, insulin sensitivity markers or body weight, although these findings are exploratory and potentially influenced by unmeasured confounders. These results should be interpreted as descriptive, within-cohort observations providing extended real-world insights into the long-term use of dolutegravir+lamivudine as a switch regimen in clinical practice.

## Supplementary Material

dkag172_Supplementary_Data

## Transparency declarations

AC received personal fees and/or speakers’ honoraria from ViiV Healthcare and Merck Sharp & Dohme. AG reports grants and/or personal fees from BMS, Gilead, Janssen, MSD and ViiV Healthcare. MM received research grants paid to institutions from Gilead Sciences and fees as speaker from ViiV Healthcare, Gilead Sciences and MSD. SR received research grants to his Institution from ViiV Heathcare, Gilead Sciences and Janssen, outside the submitted work; he was also a paid consultant for ViiV Heathcare, Gilead Sciences, Merck Sharp and Dohme, Bristol-Myers Squibb and Janssen. CM has participated in advisory boards, received study grants and/or speaker honoraria from: Abbvie, Gilead, Viiv, Janssen, Angelini, BMS, MSD. SDG was a paid consultant or member of advisory boards for Gilead, ViiV Healthcare, Janssen-Cilag, Merck Sharp & Dohme and Bristol-Myers Squibb. All other authors: none to declare.

## Supplementary data

Table S1 is available as Supplementary data at *JAC* Online.
