# Supplementary material for: ‘Leaving no stones unturned’: up to 10 years results on the effectiveness, tolerability and metabolic safety of dolutegravir+lamivudine (DTG+3TC) as a switch regimen in the ODOACRE cohort
Source: J Antimicrob Chemother. 2026 May 15;81(6):dkag172. doi: 10.1093/jac/dkag172 (PMC13175984; doi:10.1093/jac/dkag172)
Supplement: dkag172_Supplementary_Data [file dkag172_supplementary_data.docx]

Supplementary Table S1. Types of malignancies diagnosed during follow-up

| **Type of malignancy** | **N (% of all malignancies)** |
| --- | --- |
| Anal cancer | 3 (5.5) |
| Prostate cancer | 8 (14.8) |
| GI tract cancer | 5 (9.3) |
| Breast cancer | 7 (13.1) |
| Skin cancer | 9 (16.7) |
| Lung cancer | 4 (7.4) |
| Hematological malignancy | 3 (5.5) |
| Pancreatic cancer | 3 (5.5) |
| Urinary tract cancer | 4 (7.4) |
| Thyroidal cancer | 2 (3.7) |
| Other | 6 (11.1) |
